# Enhanced antitumor activity of bovine lactoferrin through immobilization onto functionalized nano graphene oxide: an *in vitro*/*in vivo* study

**DOI:** 10.1080/10717544.2020.1809558

**Published:** 2020-08-19

**Authors:** Azam Najmafshar, Mahboubeh Rostami, Jaleh Varshosaz, Dariush Norouzian, Seyed Ziyae Aldin Samsam Shariat

**Affiliations:** a Department of Clinical Biochemistry, School of Pharmacy and Pharmaceutical Sciences, Isfahan University of Medical Sciences, Isfahan, Iran; b Department of Medicinal Chemistry, School of Pharmacy, Isfahan University of Medical Sciences, Isfahan, Iran; c Department of Pharmaceutics, School of Pharmacy and Novel Drug Delivery Systems Research Centre, Isfahan University of Medical Sciences, Isfahan, Iran; d Department of Nanobiotechnology, Pasteur Institute of Iran, Tehran, Iran

**Keywords:** Bovine lactoferrin, graphene oxide, lung cancer, TC-1 cells, anticancer

## Abstract

This study aims to improve the anticancer activity of bovine lactoferrin through enhancing its stability by immobilization onto graphene oxide. Bovine lactoferrin was conjugated onto graphene oxide and the conjugation process was confirmed by FT-IR, SDS-PAGE, and UV spectrophotometry. Physical characterization was performed by DLS analysis and atomic force microscopy. The cytotoxicity and cellular uptake of the final construct (CGO-PEG-bLF) was inspected on lung cancer TC-1 cells by MTT assay and flow cytometry/confocal microscopy. The anticancer mechanism of the CGO-PEG-bLF was studied by cell cycle analysis, apoptosis assay, and western blot technique. Finally, the anticancer activity of CGO-PEG-bLF was assessed in an animal model of lung cancer. Size and zeta potential of CGO-PEG-bLF was obtained in the optimum range. Compared with free bLF, more cytotoxic activity, cellular uptake and more survival time was obtained for CGO-PEG-bLF. CGO-PEG-bLF significantly inhibited tumor growth in the animal model. Cell cycle arrest and apoptosis were more induced by CGO-PEG-bLF. Moreover, exposure to CGO-PEG-bLF decreased the phospho-AKT and pro-Caspase 3 levels and increased the amount of cleaved caspase 3 in the treated cells. This study revealed the potential of CGO-PEG as a promising nanocarrier for enhancing the therapeutic efficacy of anticancer agents.

## Background

Lung cancers are among the most common cancers and the main cause of cancer death in the world. LF, a member of the transferrin family, is an 80-kD glycoprotein with many useful biological functions including, antioxidant, antimicrobial, antiviral, anticancer, anti-inflammatory, and immunomodulatory properties (García-Montoya et al., 2012; Wang et al., 2019). Its anticancer activity has been demonstrated in several types of cancers (Tsuda et al., 2002; Jonasch et al., 2008; Yamada et al., 2008; Hayes et al., 2010; Tsuda et al., 2010; Xu et al., 2010; Li et al., 2011; Gibbons et al., 2015; Zhang et al., 2015; Arias et al., 2017). The safety and effectiveness of bovine lactoferrin (bLF) in many applications, has been approved by the European Food Safety Authority (EFSA, 2012). Compared with other anticancer agents, proteins (such as bLF) have several advantages, such as higher affinity and specificity, lower unwanted effects, and greater efficacy in the treatment of cancers. Despite the advantages, short circulation time because of enzymatic cleavage and rapid renal clearance, have been limited their applications in clinical fields. To overcome this issue, several approaches have been developed, such as PEGylation, glycosylation, and liposomal formulations. Despite these progressions, the instability of proteins in the biological environment has remained as the main challenge in clinical applications. Moreover, cancer therapy with conventional anticancer agents, have some disadvantages including nonspecific toxicity, short half-life, relative instability, needs for repeat administration, and low patient compliance. Thus, more efficient strategies are needed. These days, drug delivery systems have been attracted to more consideration in the cancer research field. A variety of nanomaterials have been used as carriers for drug delivery systems. Graphene oxide (GO) and its derivatives are among these carriers that have true two-dimensional sheets of sp^2^ carbon atoms in a honeycomb structure with unique properties. The planer structure of graphene with a large specific area and rich functional groups have made it an excellent platform for the delivery of different kinds of substances including biological molecules, anticancer agents, and other materials (Kazempour et al., 2019).

GO as a carrier for drug delivery has shown excellent biocompatibility and low toxicity in several *in vitro* and *in vivo* experiments. Because of natural nature, stability, and solubility in physiological conditions, it has been widely used in many biomedical studies (Yang et al., 2015; SreeHarsha et al., 2019). Xu and his colleagues (Xu et al., 2015) provide a conjugated form of paclitaxel onto the functionalized GO. The tissue distribution and anticancer efficacy of GO-conjugated paclitaxel were investigated in the B16 melanoma cancer-bearing C57 mice model. The GO-conjugated paclitaxel showed excellent solubility and biocompatibility. Compared with the free paclitaxel, the conjugated form indicated more circulation time as well as high tumor-targeting and tumor-suppressing efficacy. In another work, the GO loaded with SN38 showed proper biocompatibility and excellent solubility in physiological conditions (Liu et al., 2008). Immobilization of alpha-amylase onto functionalized GO was successfully constructed by Singh et al. The immobilized enzyme showed enhanced thermal and storage stability and retained 73% residual activity after 10 uses (Singh et al., 2015). A construction of R9-reduced GO loaded with paclitaxel was developed by Hashemi et al. (Hashemi et al., 2016). Their construct showed appropriate drug loading efficiency and stability in the physiologic environment. Their experiment revealed that the new compound was efficiently uptook by Hela cancer cell lines, and reduced the viability of Hela and MCF-7 cells for more than 90% after 72 h. Chitosan-functionalized graphene oxide (GO-CS), as a drug carrier, was successfully prepared by Bao et al. and used for delivery of camptothecin (CPT) to HepG2 and Hela cancer cells (Bao et al., 2011). Superior anticancer activity was obtained for the conjugated drug compared to the free drug. Besides, the carrier was also able to condense plasmid DNA into a stable complex, and the resulting construct demonstrated proper transfection efficiency in HeLa cells. In a project conducted by Zhang et al., folate targeted nano GO loaded with doxorubicin (DOX) and camptothecin (CPT) was constructed and its cytotoxicity was evaluated against human breast cancer MCF-7 cell lines (Zhang et al., 2010). Their results demonstrated that the nanocarrier containing of the two drugs specifically targeted the cells and its cytotoxicity was significantly increased in combination form compared to the use of them separately due to the synergistic effect of the drugs.

In this work, we successfully prepared a GO-based drug nanocarrier for the delivery of bLF. GO was carboxylated and then PEGylated. After that, the bLF was conjugated to CGO-PEG complex through EDC/NHS chemistry. The final construct was physiochemically characterized and its anticancer activity was evaluated against TC-1 lung cancer cells through several experiments. Moreover, its tumor growth inhibitory effects were studied in the animal model of lung cancer.

To the best of our knowledge, this is the first work that used bLF as an anticancer agent and targeting ligand simultaneously in order to improve its anticancer activity against lung cancer using the capability and advantages of PEGylated GO as a nanocarrier that enhance the protein stability and cellular uptake. Overall, the resulting construct provides a new drug delivery system that increased the therapeutic activity of bLF compared with the free form of the protein.

## Materials and methods

### Materials

Bovine lactoferrin (bLF) was obtained from Morinaga Milk Industry Company (Japan). Graphene oxide (GO, as a control), 1-ethyl-3-(3-dimethylaminopropyl) carbodiimide (EDC), N-hydroxysulfosuccinimide (NHS), fluorescein isothiocyanate (FITC), 3-(4,5-dimethylthiazol-2-yl)-2,5-diphenyltetrazolium bromide (MTT), Poly(ethylene glycol) bis(amine) (_2_HN-PEG-NH_2_, 3.4 kDa) were obtained from Sigma-Aldrich. 4-morpholinoethanesulfonic acid (MES) obtained from Biobasic Inc. (Canada). Cell culture medium (DMEM), fetal bovine serum (FBS), trypsin and penicillin/streptomycin were purchased from Invitrogen Co., USA. All antibodies including, AKT, p-AKT, GAPDH, and Caspase 3 were purchased from Santa Cruz Biotechnology (Santa Cruz, CA). TC-1 cell line and C57BL/6 mice were obtained from the Pasteur Institute of Iran. Other chemicals of analytical grade were acquired from Sigma-Aldrich Company.

### Methods

#### Synthesis of GO

GO was synthesized from graphite powder using a modified Hummer’s method (Zaaba et al., 2017). Briefly, 1 g of graphite powder, 4 ml of sulfuric acid, 830 mg of K_2_S_2_O_8_, and 830 mg P_2_O_5_ were mixed and heated at 80 °C for 4 h. After cooling at room temperature, 150 ml of deionized water was added and kept overnight on the magnetic stirrer (120 rpm, RT). After filtration, the remaining solution (pre-oxidized GO) was dried at room temperature. The pre-oxidized GO was more oxidized by the addition of H_2_SO_4_ (60 ml), KMnO_4_ (7.5 g), and water (125 ml) in an ice-water bath. After dilution with deionized water (350 ml), excess KMnO_4_ was removed by the addition of H_2_O_2_ (30 wt.%, 10 ml) and then HCl (10 wt.%, 500 ml) was added to remove the remaining of metal ions. The resulting solution was filtered and then washed with deionized water for several times. To achieve nanosized and single-layered GO, the suspension was dispersed by probe sonication (SONOPLUS, BANDELIN electronic, GmbH). The resulting solution was freeze-dried (Christ, Alpha 1-2 LDPLUS, Germany) to obtain brown GO powder.

#### Carboxylation of GO

For the preparation of carboxylated GO (CGO), GO was oxidized under the strongly basic condition to convert hydroxyl, epoxide, and ester groups to carboxylic acid moieties. To do this, 100 mg of GO was dissolved in deionized water (DW) and completely dispersed by probe sonication for about 2 h at room temperature (RT). Chloroacetic acid (5 g) and NaOH (6 g) was added and the solution was sonicated for another 3 h at RT. The resulting CGO solution was centrifuged and washed several times with DW. Then, the solution was dialyzed (cut off: 12.4 kDa) against DW for 5 days to remove the extra reagents. The final pure product was dispersed in DW by probe sonication for 1 h and kept at RT.

#### PEGylation of CGO

CGO was PEGylated in order to improve its solubility and stability in physiological and environmental conditions. To do this, the amine groups in NH_2_-PEG-NH_2_ were conjugated to carboxyl groups in CGO. Briefly, the CGO (1 mg/ml, 50 ml) was probe-sonicated (100 W, 1 h) to obtain a homogenous solution and then mixed with EDC (100 mg) for 10 min and followed by addition of NHS (60 mg). The solution was mixed for 2 h on a magnetic stirrer (120 rpm, RT), then added dropwise to the NH_2_-PEG-NH_2_solution (250 mg in 5 ml DW) and stirred overnight. Extra excipients were removed by dialysis (cut off: 12.4 kDa) against DW for 4 days. The final solution (CGO-PEG) washed several times with DW (9000 × *g*, 20 min, RT) and then characterized by FTIR spectroscopy.

#### Conjugation of bLF to CGO-PEG

Bovine LF was conjugated onto CGO-PEG using EDC/NHS coupling chemistry. Briefly, bLF was dissolved in 50 mM MES buffer (1 mg/ml, 20 ml) and mixed with EDC (50 mg) for 10 min. After that, the NHS (30 mg) was added to the mixture and stirred for 60 min. The solution was filtered and washed several times with cold DW to remove the excess agents. The sediment was dispersed in 30 ml of MES buffer (50 mM) and mixed with CGO-PEG solution (1 mg/ml, 2.5 ml) for 6 h. The final product (CGO-PEG-bLF) was filtered and washed several times by filtration device (Millipore, Amicon Ultra-4, 100 kDa), characterized by FTIR spectroscopy and kept in 4 °C before use. The attachment of bLF to the carrier was confirmed by SDS-PAGE and the amount of conjugated protein was determined by the Bradford method.

### Characterizations

Particle size, zeta potential, and size distributions of GO and its related compounds were analyzed by photon correlation spectroscopy using a Zetasizer instrument (ZEN3600, Nanoseries, Malvern, UK). Shape and surface morphology was analyzed using atomic force microscopy (AFM). For AFM analysis, 20 µl of the sample was placed on mica slide, air-dried, and visualized under the Nanowizard II AFM (JPK Instruments, Germany) microscope. The images were acquired in contact mode under ambient conditions. The chemical composition and the surface functional groups were analyzed by Fourier transform infrared spectroscopy (FTIR, Thermo-Nicolet spectrometer) with KBr pellets in the range of 400–4000 cm^−1^. UV-Vis spectrometer (Hitachi Inc., U-2910) was used to measure the optical absorption properties of GO and related products.

#### Cell culture

The mouse lung cancer TC-1 cells were supplied by the National Cell Bank of the Pasteur Institute of Iran. The cells were cultivated at 37 °C under a humidified 5% CO_2_ atmosphere in DMEM medium supplemented with 10% fetal bovine serum and 100 uints/ml of penicillin and 100 µg/ml of streptomycin.

#### Cell viability study

The cytotoxic effects of free bLF and its corresponding conjugates with GO were studied by 3-(4,5-dimethylthiazol-2-yl)-2,5diphenyltetrazolium bromide (MTT) assay (Chiani et al., 2017). Briefly, the TC-1 cells were seeded at 1 × 10^4^ cells in each well of 96-well plates. After 24 h, the cultured cells were treated with different concentrations (1.78–56.98 µM) of free bLF, CGO-PEG-bLF, and their controls (PBS, CGO-PEG) for 48 h. Then, 20 μl of MTT solution (5 mg/ml in PBS) was added to each well and the cells were incubated at 37 °C for another 4 h. After that, the medium was discarded and 150 µl of DMSO was added to dissolve the insoluble formazan. The absorbance values were measured using a microplate reader (AccuReader, M965 Series, Metertech, Taiwan) at 570 nm to determine the viability of the cells. The percent of cell viability was calculated by the following formula: (mean OD of treated group/mean OD of control group) × 100.

#### FITC-Labeling of bLF and CGO-PEG-bLF

The bLF and CGO-PEG-bLF was labeled with FITC in order to be used in cellular uptakes and cell cycle analysis. Briefly, bLF and CGO-PEG-bLF (0.3 mg protein) were mixed with fluorescein isothiocyanate (FITC, 1 mg/ml in DMSO) and bicarbonate buffer (1 ml, pH 10) for 2 h in a dark place, and then filtrated (Millipore, Amicon Ultra-4, 10 kDa) to remove excess unbound FITC and washed (7000 × *g*, 20 min, 4 °C) several times by PBS (pH 7.4).

#### Cellular uptake efficiency

The TC-1 cells were seeded in 12-well plates (or seeded on coverslips for confocal microscopy analysis) at a density of 2 × 10^5^ cells per well and were allowed to adhere for 24 h. Then, the medium was replaced with a fresh medium containing free bLF and CGO-PEG-bLF at the same concentration of protein (28.5 µM). After incubation for 3 h, the cells were washed with PBS, fixed (30 min at RT in a dark place) with 3 ml of cold and fresh 3.5% paraformaldehyde, and then qualitatively analyzed by confocal electron microscopy (TCs SP5 II, Leica Microsystems Co., USA). For quantitative analysis, the cells were collected, washed, and suspended in 1 ml of PBS, and analyzed by flow cytometry (Partec GmbH, Munster, Germany) using Flowjo Software (FlowJo, LLC, Ashland, OR).

#### Cell apoptosis assay

To detect and quantify apoptosis in TC-1 cells induced by the free bLF and CGO-PEG-bLF, the cells were stained with Annexin V-FITC kit (BioVision Inc., Milpitas, CA, USA), according to manufacturer’s protocol. Briefly, TC-1 cells were seeded at a density of 5 × 10^5^ in each well of 6-well culture plates and incubated for an overnight at 37 °C in 5% CO_2_. Then, the old medium was discarded, and fresh medium containing 10 µM of free bLF and CGO-PEG-bLF was added and the cells were incubated for 48 h. After that, the medium was discarded and the cells were collected and washed twice by PBS. The cells were suspended in 500 µl of 1X binding buffer at a concentration of 5 × 10^5^ cells/ml. And then added 5 μl of Annexin V-FITC and 5 μl of propidium iodide (PI) solution, gently vortexed and incubated for 5 min in the dark at RT before analysis by flow cytometry (CyFlow SL, Partec, Germany). Data analysis was performed by FlowJo software.

#### Cell cycle analysis

This study was performed to inspect the effect of free bLF and CGO-PEG-bLF on cell cycle patterns. To do that, TC-1 cells were seeded at a density of 5 × 10^5^ in each well of 6-well culture plates and incubated for 24 h at 37 °C in a humidified atmosphere containing 5% CO_2_. Then, the old medium was replaced with fresh medium containing 10 µM of free bLF or CGO-PEG-bLF as triplicates and the cells were incubated for 48 h. Afterward, the medium was discarded and the cells were harvested by trypsin-EDTA, washed twice by cold PBS, and fixed by 70% cold ethanol and placed on ice for at least 2 h and then collected by centrifugation (200 × *g*, 6 min). The cell was washed and resuspended in PBS containing 20 µg/ml propidium iodide (PI), 1 mg/ml RNase A and 0.1% Triton X-100 and incubated at RT for 30 min in a dark place. The DNA contents and cell proportions in every phase of the cell cycle were analyzed by a flow cytometer (CyFlow SL, Partec, Germany) using FlowJo Software. In this analysis, each sample contained at least 40,000 single cells, and appropriate gating was employed to exclude cell debris from the analysis.

#### *In vitro* release study

To quantify the release profile of bLF from CGO-PEG-bLF complex, 1 mg of the complex was added to 10 ml of PBS buffer with two different pH values (pH 7.4 as physiological pH, and pH 5.2 was selected as tumor microenvironment pH) and placed in shaking incubator (37 °C, 120 rpm, 72 h). At different interval times, 200 µl of buffer was taken and replaced with fresh buffer. The amount of released protein from the complex was measured by Bradford assay and estimated according to the standard curve that previously provided by serial dilution of BSA (Onishi et al., 2007).

#### Western-blot assay

To elucidate the mechanism of action of free bLF or CGO-PEG-bLF in TC-1 cells, the expression of the major proteins involved in its biochemical pathways were studied by western blot analysis. To do this, 1 × 10^6^ cells/well of 6-well plates were seeded and incubated at 37 °C in a humidified atmosphere containing 5% CO_2_. After 24 h, the old medium replaced with fresh medium containing 10 µM of free bLF and CGO-PEG-bLF, and the cells were incubated for 24 h and 48 h. Subsequently, the cells were washed three times with cold PBS and lysed for 60 min (by several vortex) on ice with 100 μl of RIPA Lysis Buffer (20 mM Tris-HCl pH 7.5, 0.5% Nonidet P-40, 0.5 mM PMSF, 100 mM β-glycerol 3-phosphate, and 0.5% protease inhibitor cocktail). The lysates were centrifuged at 12,000 × *g* for 10 min at 4 °C, and the supernatants were collected as the total cellular protein. Protein contents were measured by Bradford assay. Protein (30 mg) from each sample was loaded on 10% SDS-PAGE and transformed onto a PVDF membrane. To avoid nonspecific interactions, the blots were blocked with 5% fat-free dry milk dissolved in PBST buffer (PBS containing 0.1% Tween-20) and stirred at RT for 2 h. Then, the membranes were incubated with the primary antibodies (1:500) overnight at 4 °C. After that, the membranes were washed twice with PBST and incubated with an HRP-conjugated secondary antibody (1:500) for about 2 h. Finally, after washing with PBST buffer, the chemiluminescence of the membranes was detected by ECL detection reagent (Millipore-Merck) in a ChemiDocTM XRS system (Bio-Rad). The GAPDH, a housekeeping protein, was used as the control for normalization.

#### Animal study

Female C57BL/6 mice (5 to 6-weeks-old, 18–20 g) were obtained from the Pasteur Institute of Iran. All animal experiments were performed in accordance with the institutional ethics committee regulations and guidelines. Mice were maintained in a 12 h light/dark cycle at a constant temperature of 24 ± 1 °C with free access to food and water *ad libitum*. The tumor model of lung cancer was generated by subcutaneous injection of 2 × 10^6^ of the TC-1 cells in 100 µl PBS in the flank of the mice (Amiri et al., 2018). When tumor size was reached to about 100 mm^3^, the mice were randomly divided into 4 groups (8 mice per group) for subsequent experiments. The mice in each group were treated (3 times, once every 3 days) with free bLF (50 mg bLF per Kg/body weight), CGO-PEG-bLF (containing 50 mg bLF and 4.35 mg GO per Kg/body weight), CGO-PEG and PBS, respectively. The animal weighs and tumor sizes were measured with a digital balance and a digital caliper every week. The tumor volume was calculated as the volume (mm^3^) = (tumor width)^2^ × (tumor length)/2. For pathology and histological analysis, the solid tumor was fixed in 10% formalin, embedded in paraffin and sectioned, stained with hematoxylin and eosin (H&E), and studied under a light microscope. Median survival time (MST) and the increased life span in each group of mice was calculated according to the following equation:

(1) ILS% = [(MST of treated mice/MST of control mice) – 1] × 100

Where MST means the time (day) at which half of the animals had died.

### Statistical analysis

Data analysis was performed with GraphPad Prism v8 Software. Data were analyzed by *t*-test and analysis of variance (ANOVA); when statistical differences were detected, Tukey’s Multiple Comparison test was performed. Data were expressed as mean ± SD of at least three independent experiments and a value of *p* < .05 was considered significance.

## Results and discussion

### Characterization

**Figure 1.**
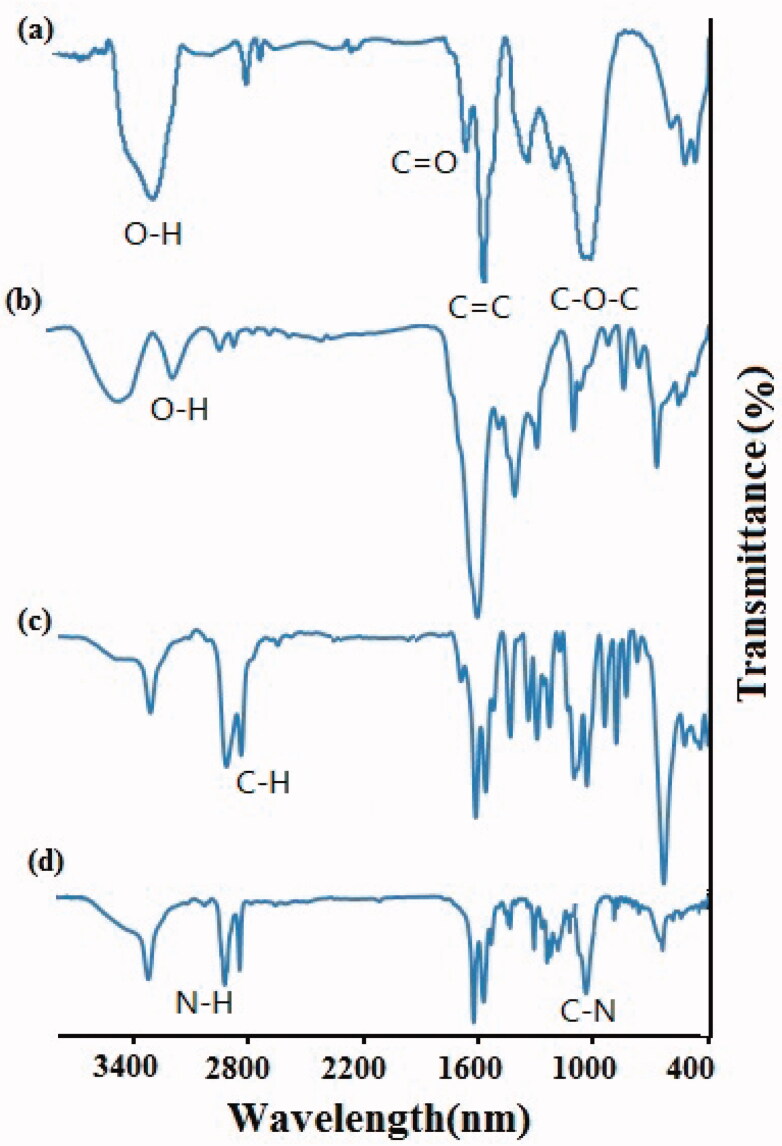
FTIR spectra of GO (a), CGO (b), CGO-PEG (c) and CGO-PEG-LF (d).

In this study, GO was prepared by a modified Hummers method and subsequently carboxylated, PEGylated, and conjugated with bLF as mentioned above. To improve the solubility, stability, and biocompatibility of GO in physiological conditions, its conjugation with polyethylene glycol, as a hydrophilic polymer, was performed. To do this and for enhanced PEGylation yield, the hydroxyl and epoxy groups of the GO were converted to carboxyl groups, by which GO could conjugate with an amine group of NH_2_-PEG-NH_2_through amidation (formation of amide bonds). PEGylated GO was then covalently conjugated with bLF through interaction between carboxyl groups of the protein with free amine groups of PEG molecules. The amount of conjugated bLF on GO was calculated by Bradford assay and was obtained around 0.57 mg/ml in the final product. The successful carboxylation, PEGylation, and conjugation of GO with protein were confirmed by FTIR, DLS, UV-Vis and AFM methods. The results of FTIR analysis was indicated in Figure 1.

In FTIR analysis of GO, the adsorption peaks around at 3400, 1720, 1620, and 1100 cm^−1^ are related to hydroxyl (-OH), carboxyl (C=O), aromatic (C=C) and epoxy (C–O–C) bonds, respectively (Lu et al., 2020). After the carboxylation of GO, the resulting derivative (CGO) showed stronger absorption band at 1620 cm^−1^, a decrease in hydroxyl bond around at 3400 cm^−1^, and an increase in hydroxyl bond around at 2800–3200 cm^−1^ which confirmed the formation of extra carboxyl groups on the surface of GO. Furthermore, a reduction in epoxy peaks (C–O–C) at 1100 cm^−1^ which observed in CGO possibly attributed to the synergistic effects of the carboxylation process on both epoxide and hydroxyl groups (Zhang et al., 2011). Successful PEGylation of GO could be verified by the peaks approximately at 2850 cm^−1^ (C–H bond), 1650 cm^−1^ (NH–CO or amide bond), and 1100 cm^−1^ (C–O–C bond), respectively. By PEGylation and formation of an amide bond between carboxyl groups of GO and amine groups of NH_2_-PEG-NH2, the sharp peak at 1720 cm^−1^ of GO was decreased and shifted to a peak around 1650 cm^−1^ (–NH–CO–), which confirm the decreasing of many oxygen groups, the formation of amide groups and successful PEGylation (Wang et al., 2012; Chen et al., 2014; Xu et al., 2014). The peaks approximately at 1240 cm^−1^ (C–N), 1430 cm^−1^ (C–C), 2950 cm^−1^ (N–H), and 3350 cm^−1^ (O–H), confirmed the conjugation of protein on the surface of GO.

**Figure 2.**
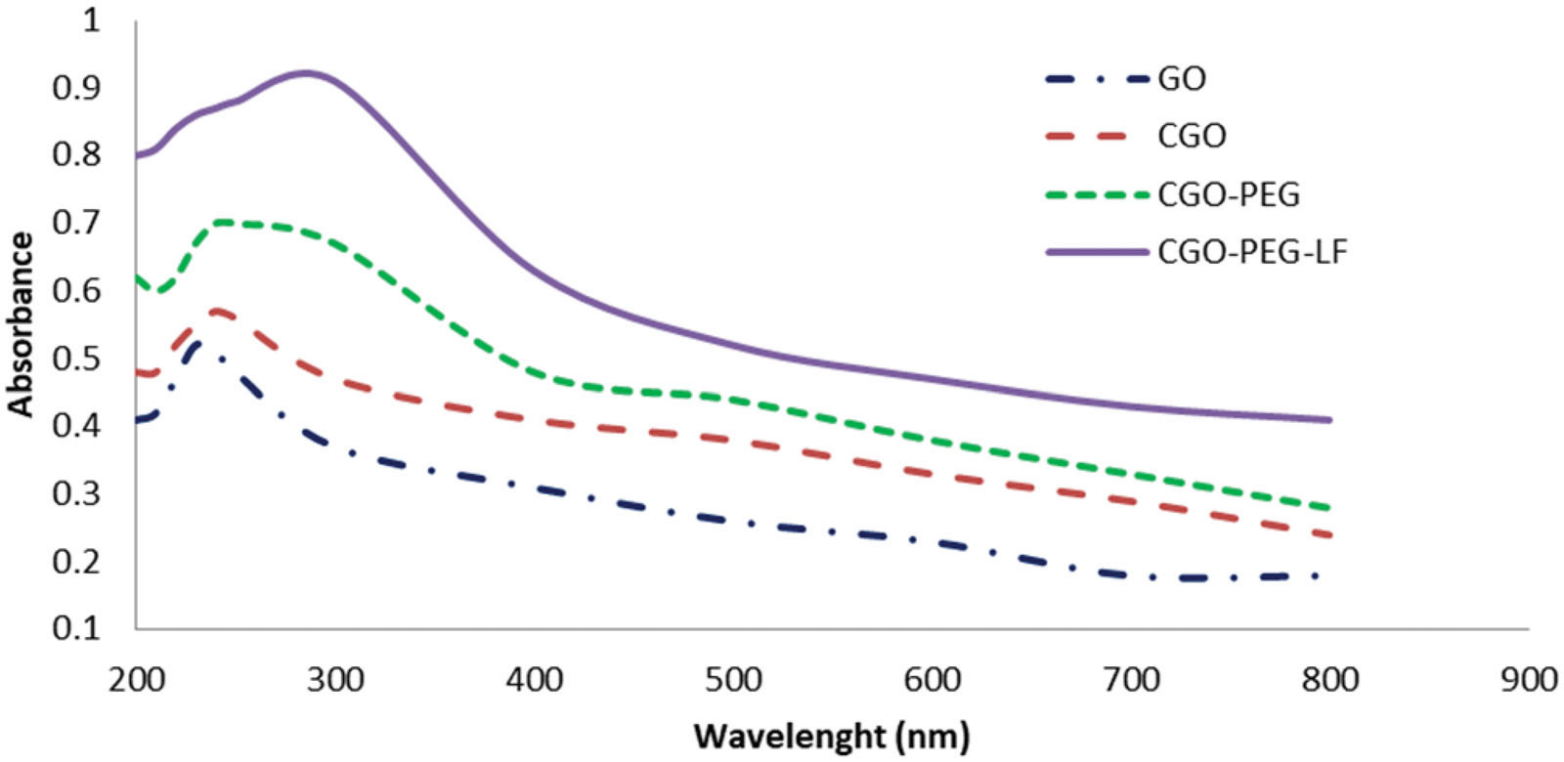
UV-Vis spectrophotometry of GO and its derivatives. Maximum absorbance for CGO-PEG-LF was obtained in range of 250–280 nm.

According to the UV-vis spectra (Figure 2), the maximum optical density of GO was obtained around 230 nm which is a characteristic peak of GO that was assigned to the π–π* transition of C=C, while a shoulder peak near to 300 nm corresponded to *n*–π* transition of C=O bonds. By PEGylation and conjugation with lactoferrin, the UV-vis spectra of GO was relatively changed. So, the near-infrared and visible absorbance of GO was increased and the maximum absorption was obtained around 250–280 nm. The increase of optical density could be related to the hydrolysis of the ester bond and opening of epoxide groups of GO during the carboxylation process. Furthermore, this evidence confirmed the existence of bLF on the surface of GO that showed maximum absorbance at 280 nm due to the presence of aromatic amino acids in the structure of the protein.

**Figure 3.**
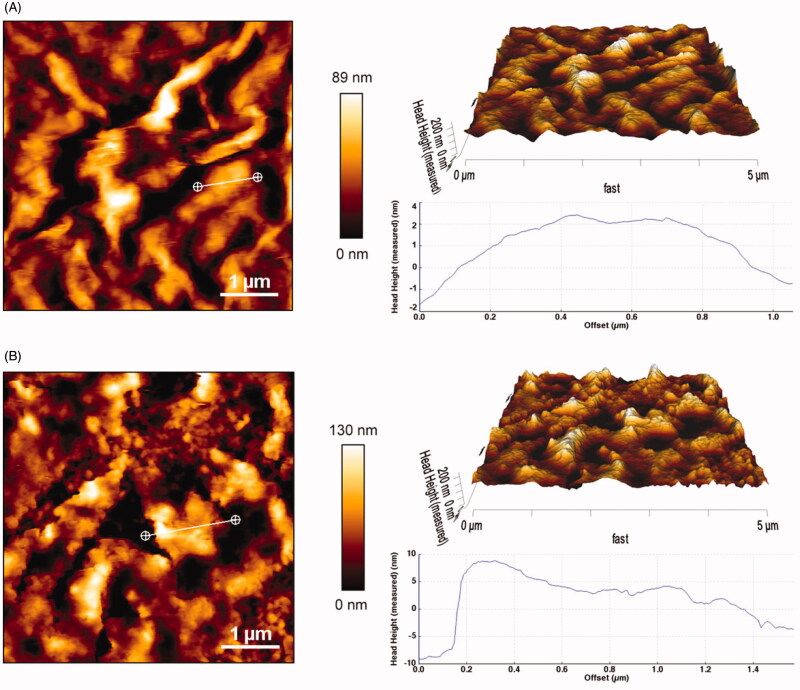
AFM images of GO (A) and CGO-PEG-LF (B). The images were taken in contact mode. Sharp edges and smooth surface with thickness of 1–2 nm was obtained for GO whereas the CGO-PEG-LF showed coarse edges and rough surface with thickness of 4–10 nm.

The morphology of GO and CGO-PEG-bLF was visualized by AFM, as shown in Figure 3, the thickness of GO was obtained around 1–2 nm (single-layered) with a relatively smooth surface and sharp edges, which was accordance to the previous reports (Stankovich et al., 2006; Li et al., 2008; Emadi et al., 2017), but it was obviously increased to 4–10 nm and become more roughly with coarser edges after PEGylation and conjugation with bLF that confirmed the successful formation of CGO-PEG-bLF. The lateral size of the GO and CGO-PEG-bLF was obtained around 89–130 nm, respectively; which is a suitable size for a drug carrier.

The results of DLS analysis have presented in Table 1. As shown in this table, the mean particle size of GO and the final construct was obtained around 95 and 153 nm which is in the optimum range for a drug delivery system (Li et al., 2010; Akbari et al., 2020). As illustrated, the mean size of GO and CGO-PEG-LF obtained from AFM is seemingly smaller than that measured by DLS analysis. This difference may be attributed to the hydrodynamic effect of the aqueous layer that covers the vesicles and relatively enhanced the size of the particles measured by the DLS technique. By the addition of PEG and then bLF, the size of the GO was increased significantly and its zeta potential became more positive. Increasing the size and zeta potential confirmed the successful PEGylation and conjugation of LF on the surface of the GO. Carboxylation of GO decreased its surface charge because of the negative charge of carboxyl groups. In contrast, PEGylation with NH2-PEG-NH_2_positively increased the zeta potential of GO due to the positive charge of amine groups of PEG molecules. By conjugation to bLF, the positive charge of the GO was dramatically increased and reaches to about −9.2 ± 1.7 mV. This increase in zeta potential could be related to the positively-charged nature of the protein (Qiang et al., 2020).

**Table 1.** Characterization of GO and its derivatives using DLS analysis.

Formulation	Size (nm)	Zeta potential (mV)	PDI
GO	95.3 ± 1.7	−33.3 ± 2.3	0.31 ± 0.03
CGO	100.2 ± 2.1	−43.1 ± 2.1	0.24 ± 0.04
CGO-PEG	123.7 ± 3.6	−27.8 ± 2.3	0.22 ± 0.04
CGO-PEG-bLF	153.4 ± 4.8	−9.2 ± 1.7	0.38 ± 0.07

Results are presented as mean ± SD (*n* = 3).

### *In vitro* cell viability assay

**Figure 4.**
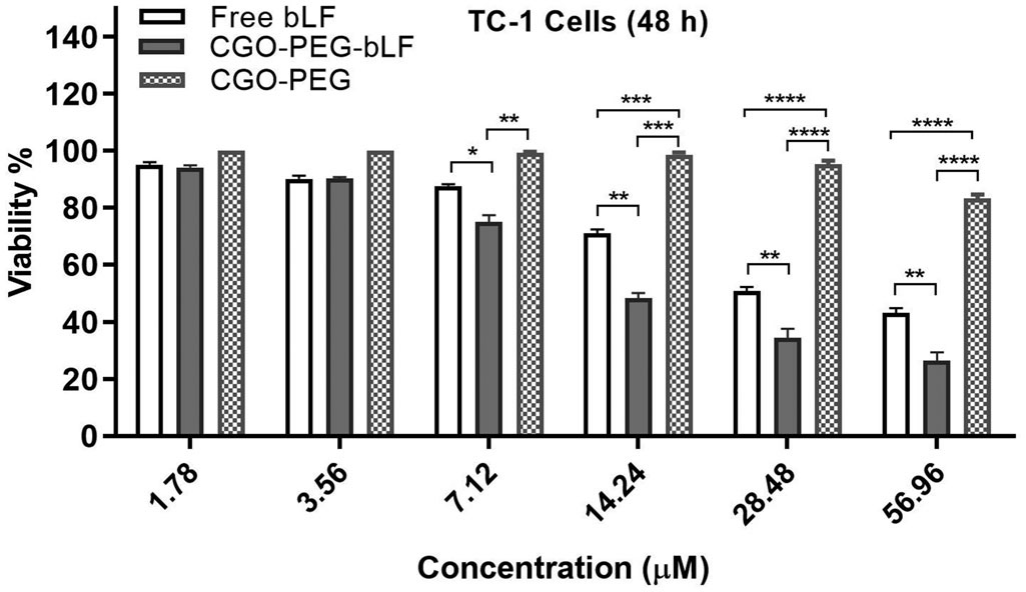
Effect of free bLF, CGO-PEG-bLF and its control (CGO-PEG) on the viability of TC-1 cells. Results are expressed as mean ± SD of 3 independent experiments (*p < .05, **p < .01, ***p < .001 and ****p < .0001).

The cytotoxic effect of free bLF and CGO-PEG-bLF was investigated on lung cancer TC-1 cells by MTT assay. As shown in Figure 4, CGO-PEG-LF showed significantly higher cytotoxicity compared to free bLF. Both free bLF and its conjugated form showed significant cytotoxicity against the cells in a dose-dependent manner. The half-maximal inhibitory concentration (IC50) for free bLF and CGO-PEG-bLF were obtained around 28.62 ± 2.38 µM and 11.05 ± 1.28 µM, respectively. It means that with the same bLF concentration, the anticancer activity of CGO-PEG-bLF against TC-1 cells is approximately 2.5-fold higher than that of free bLF. The increased cytotoxicity can be attributed to the synergistic effect of CGO-PEG as a nanocarrier to improve cellular uptake of bLF through enhanced permeability and retention (EPR) effect. No considerable cytotoxicity (about 20%) was detected for CGO-PEG (control) even at the maximum concentration that used in the study (400 µg/ml), suggesting that this carrier was not cytotoxic by itself and did not significantly impact on the cytotoxicity of bLF against the cancer cells. It should be noted the dosage of CGO-PEG adopted in our experiments was blew the 100 µg/ml. No cytotoxicity of CGO-PEG may be related to the optimum amount of CGO-PEG and the existence of PEG molecules in the structure of formulation that improves the biocompatibility and decreasing the cytotoxicity of the complex. Moreover, the cell type and exposure time may be effective in the cytotoxicity of the carrier.

### Cellular uptake study

**Figure 5.**
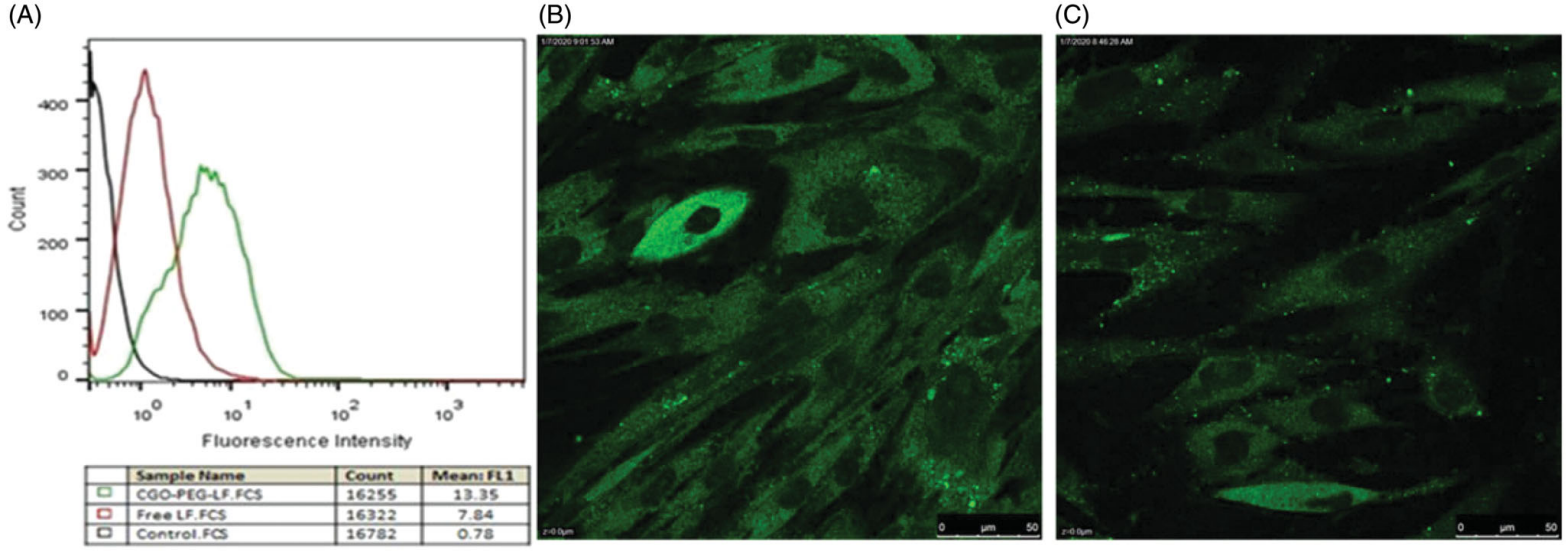
Cellular uptake of free bLF and its conjugated form (CGO-PEG-bLF) by TC-1 cells. The experiment was studied by flow cytometry (A) and confocal electron microscopy (B, C). (B) The cell treated by CGO-PEG-bLF and (C) the cells treated by free bLF.

Cellular uptake of CGO-PEG-bLF and free bLF was studied by flow cytometry (quantitatively) and confocal electron microscopy (qualitatively). The results of the experiment showed that in both methods, a greater amount of CGO-PEG-bLF has been taken up by the TC-1 cells (Figure 5). This fact clearly demonstrated that CGO-PEG facilitates and enhanced the penetration of the bLF across the cell membranes (Xu et al., 2015). The higher uptake of CGO-PEG-bLF respect to free bLF was in agreement with the results of cytotoxicity in TC-1 cells that showed significantly more cytotoxic effect for CGO-PEG-bLF compared to free protein.

### Apoptosis study

**Figure 6.**
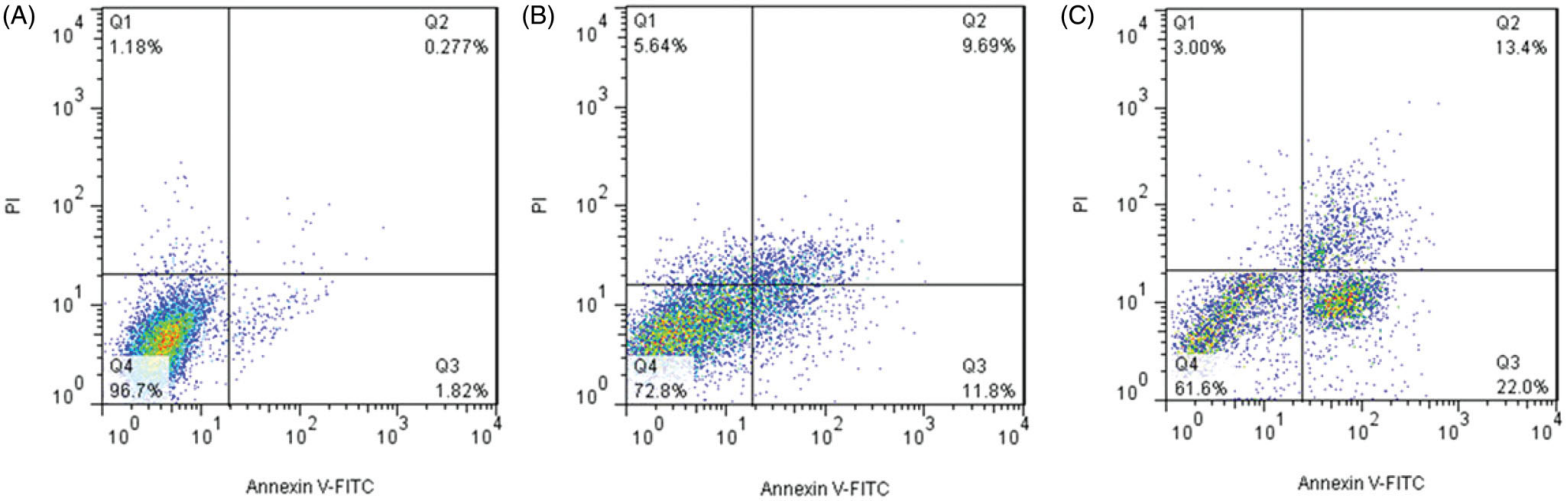
Dot plots of flow cytometric analysis to study the effect of free bLF and CGO-PEG-bLF to induce apoptosis in TC-1 cells after 48 h treatment. (A) The cells treated by control (CGO-PEG), (B) the cells treated by free bLF, and (C) the cells treated by CGO-PEG-bLF.

A decrease in cell proliferation can be related to inhibition of cell division or induction of cell apoptosis. Induction of apoptosis is the main mechanism of anticancer agents to inhibit the growth of cancers (Dasari et al., 2018). To address this issue, the ability of free bLF and CGO-PEG-bLF to induce apoptosis was evaluated by Annexin V-FITC staining assay. The results of this experiment were illustrated in Figure 6. The results showed that treating the TC-1 cells with free bLF or CGO-PEG-bLF led to increasing the percentage of early (AV+/PI−) and late apoptotic cells (AV+/PI+) after 48 h of incubation compared with control. The total percentage of early and late apoptotic cells in CGO-PEG-bLF, free bLF, and control was obtained 35.3 ± 2.6%, 25.4 ± 1.3%, and 2.2 ± 0.4%, respectively. The induction of cell apoptosis by CGO-PEG-bLF was significantly (*p* < .05) higher than that of free bLF. According to these data, cell growth inhibitory effect of bLF and CGO-PEG-bLF can be attributed to its role in the induction of apoptosis. This result was in agreement with the results of cell cycle analysis in which a higher level of sub-G1 cells, as an index of apoptosis, was observed for the treated cells (Villanueva et al., 2018). The induction of apoptosis by bLF was reported in several studies. This effect was mainly dependent on the cell type, dose of treatment, and time of the experiment and involved with different signaling pathways (Zhang et al., 2014; Chea et al., 2018; Guedes et al., 2018).

### Cell cycle analysis

**Figure 7.**
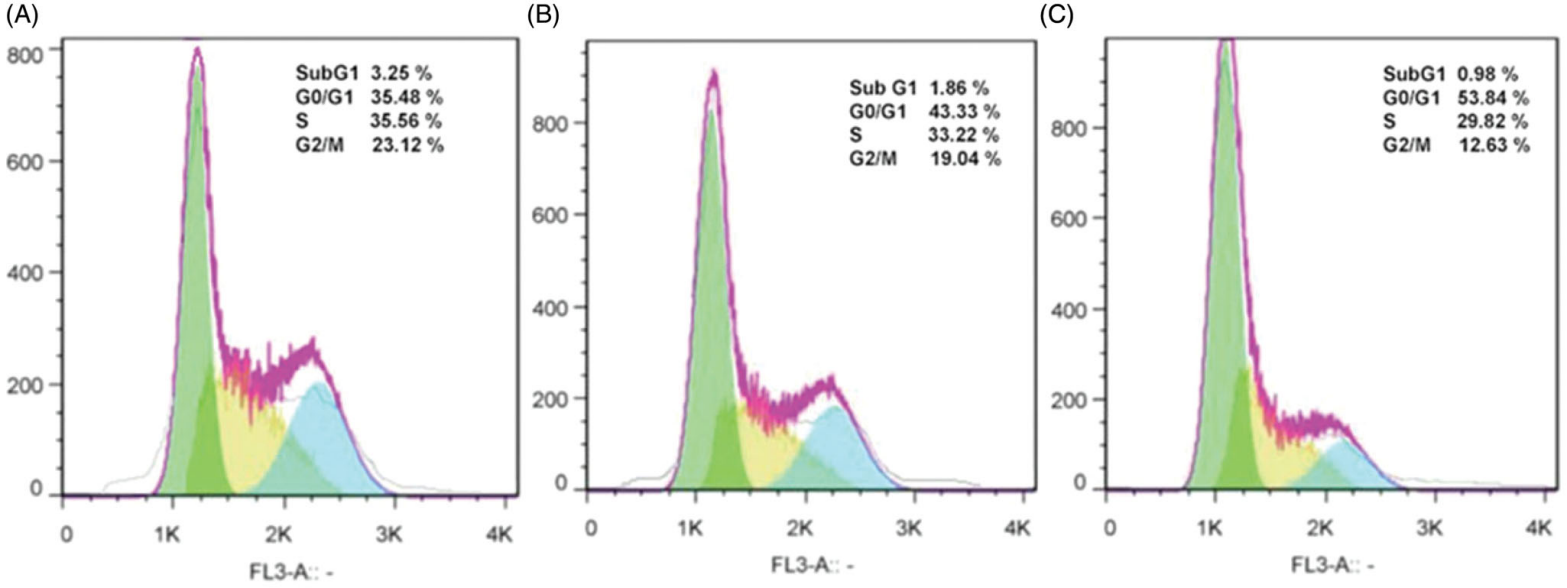
Cell cycle analysis of TC-1 cells treated by CGO-PEG-bLF (A), free bLF (B), and the control (C) for 48 h by the same amount of protein concentration (10 µM).

Cell cycle arrest is one of the main mechanisms by which anticancer agents inhibit the growth of cancer cells. The effect of free bLF and CGO-PEG-bLF on cell cycle progression was studied by flow cytometry. As presented in Figure 7, the representative cell cycle distribution images indicated that exposure to CGO-PEG-bLF, induce more growth inhibitory effect on the cell cycle progression for the TC-1 cells compared to free bLF. Compared with the control, the proportion of cells at G2/M phase increased in both free bLF and its conjugated form; but, the effect of CGO-PEG-bLF in arresting the cells was significantly higher than that of free bLF. Despite our results, several reports have demonstrated that bLF arrested the cell cycle in the G1/S phase in some types of cancers (Zhou et al., 2008; Xu et al., 2010; Chea et al., 2018). Induction of cell cycle arrest at the G2/M phase in MDA-MB-231 and T-47D cells has been reported by Zhang et al. which was in agreement with our findings (Zhang et al., 2014). It seems that this variation in the function of bLF in disrupting cell cycle progression pattern is cell type- or treatment time-dependent and may be affected by the bLF concentration and genetic background of the cells. Besides, the percentage of the hypodiploid subpopulation cells (sub-G1) was significantly increased in the cells treated by free bLF and CGO-PEG-bLF compared to control. Although, the increment of sub-G1 fraction in CGO-PEG-bLF treated cells was much more than that of free bLF. Increased percentage of sub-G1 cells as an index of apoptotic cells was in agreement with the results of apoptosis study.

### *In vitro* release study

**Figure 8.**
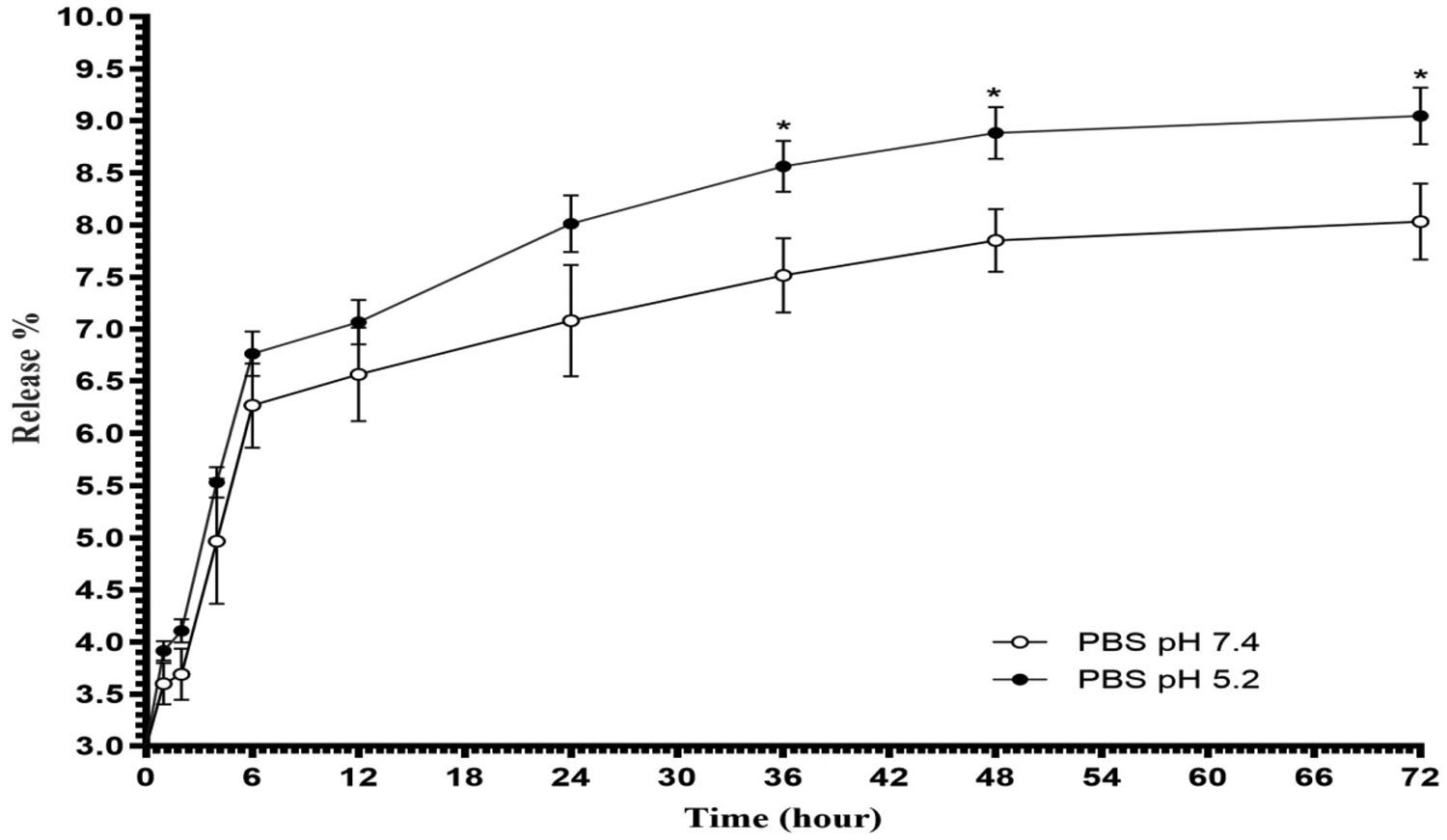
Release profiles of bLF from CGO-PEG-bLF complex in two different pH values at 37 °C. *p < .05. Each points represents the mean ± S.D. (*n* = 3).

*In vitro* release profile of bLF from CGO-PEG-bLF construction at two different pH values (5.2 and 7.4) was illustrated in Figure 8. As indicated, in both pH values, the initial burst release during the first 6 h was followed by a slow and sustained release rate. In physiological condition (pH 7.4), the release of bLF was about 6.26 ± 0.4% at the burst phase and then became slow and remained constant till the end of the experiment and reached to 8.03 ± 0.36% after 72 h. The same release profile was obtained at pH 5.2 (simulate the pH of cancer cells) with a significantly faster rate so that the total release of bLF was reached to 9.05 ± 0.47% at the end of the experiment. A similar biphasic profile of release was reported before (Pan et al., 2019). The burst effect of release is probably attributed to superficially attached bLF molecules. *In vitro* release study revealed that CGO-PEG-bLF had excellent stability in both pH values, in which a very low amount of bLF was released from GO-PEG-PTX complex within 72 h. The low release of bLF keep its effective blood concentration for a long time and let it more time to reach the target and exert its therapeutic effects.

### Western blot analysis

**Figure 9.**
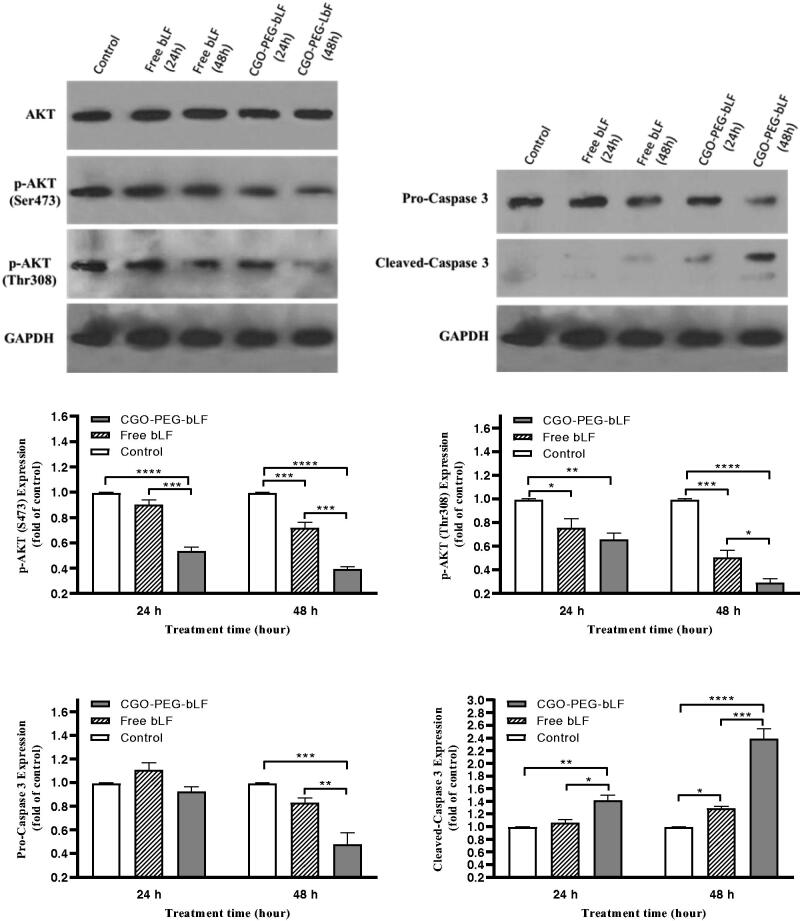
Expression of apoptosis-related proteins (AKT and caspase 3) in TC-1 lung cancer cells exposed to 10 µM of protein as free bLF and/or CGO-PEG-bLF for different times. The protein levels of phospho (p)-AKT, pro-caspase 3 and cleaved caspase 3 in the cells were analyzed using Western blot. GAPDH was used as house-keeping protein. Results are expressed as mean ± SD (*n* = 3).

Bovine LF has been demonstrated to induce apoptosis in many types of cancers through upregulation and downregulation of several proteins including caspases and AKT proteins. Western blot analysis was performed to understand the mechanism of action of bLF in inducing apoptosis. For this purpose, the protein expression of AKT, p-AKT, Caspase 3, pro-Caspase 3, and GAPDH (house-keeping protein, control) were determined at 24 and 48 h post free bLF and CGO-PEG-bLF treatment. The results indicated that both free and CGO-PEG-conjugated bLF could significantly downregulate the protein expression levels of p-AKT (Ser473 and Thr308) and pro-Caspase 3 (Figure 9). In contrast, the protein level of cleaved caspase 3 was significantly increased in TC-1 treated cells. Although a stronger effect was obtained for CGO-PEG-bLF compared to free bLF. Furthermore, these events occurred in a pronounced time-dependent manner. So, by increasing the exposure time, the upregulation or downregulation of the mentioned proteins were increased. There were no changes in the expression of GAPDH in the treated cells. These findings demonstrated that cell growth inhibitory effects of free bLF and CGO-PEG-bLF are possibly mediated by AKT and caspase 3 signaling pathways. Although, other proteins may be involved in this process. Similar results regarding effect of bLF on the three cell lines of oral squamous cell carcinoma (HSC1, HSC2, HSC3) have been reported by Chea et al (Chea et al., 2018). Their findings indicated that bLF downregulated the phosphorylation of AKT and upregulated the cleaved-caspase 3 protein selectively in HSC3 cells but not in RT7 (normal human oral keratinocytes).

### Antitumor efficacy in an animal model

**Figure 10.**
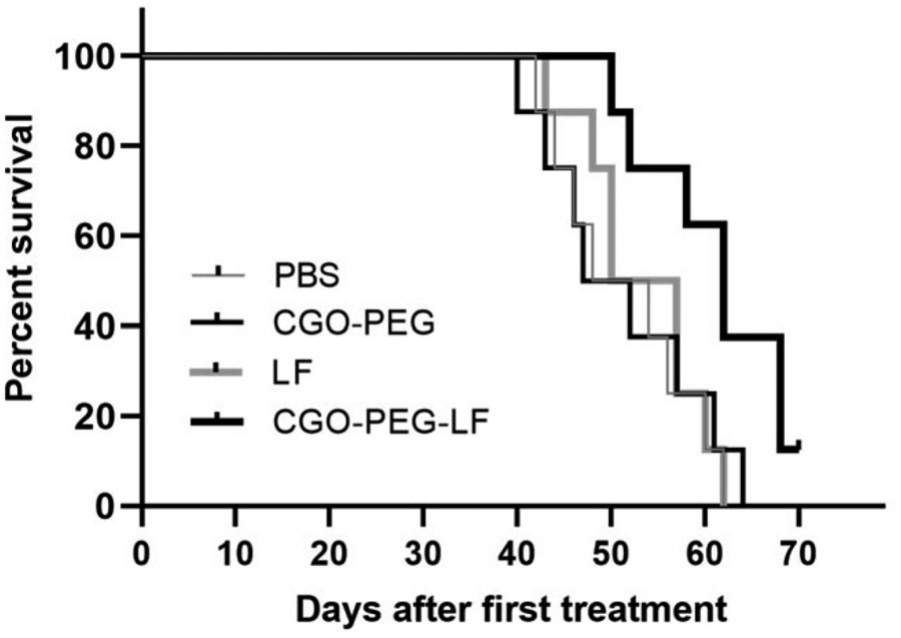
Kaplan–Meier survival curve of lung tumor-bearing mice i.v. injected by equal amount of bLF (50 mg/Kg, every three days for a total of 3 times) in form of CGO-PEG-bLF or free bLF. Control groups were treated by the equal volume of the tests (100 µl).

**Figure 11.**
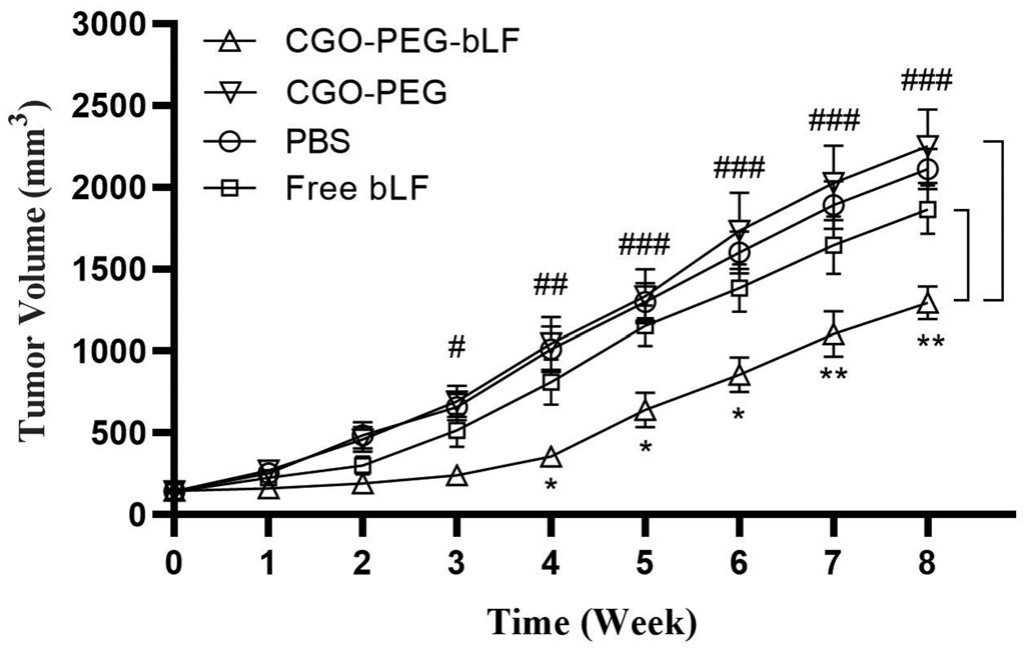
Tumor growth inhibitory effects of CGO-PEG-bLF, free bLF and related controls after 3 times (once every 3 days) intravenous injection with 50 mg protein/Kg in form of free bLF or CGO-PEG-bLF to the lung cancer bearing mice. (CGO-PEG-bLF vs CGO-PEG (control: #*p* < .05, ##*p* < .01, ###*p* < .001; and CGO-PEG-bLF vs free bLF: *p < .05, **p < .01). Data presented as mean ± SD.

Tumor inhibitory effect of free bLF and CGO-PEG-bLF was studied on TC-1 lung tumor bearing- C57BL/6 mice. The median survival time (MST) of the mice treated with CGO-PEG-bLF was increased significantly compared to free bLF and controls (PBS and CGO-PEG groups), and their MST levels of CGO-PEG-bLF, free bLF, PBS and CGO-PEG were obtained around 62, 52.5, 51, and 49.5 days, respectively. The MST levels in the free LF and control groups were not obviously different from each other and no significant tumor-suppressing effect or longer survival time was obtained for the free bLF group. Moreover, the increased life span (ILS%) of the mice in CGO-PEG-bLF and free bLF groups was increased by approximately 25 and 5% with respect to their controls (Figure 10). It meant that the ILS% in the mice treated by CGO-PEG-bLF was 5-fold higher than that of free bLF. As illustrated in Figure 11, the CGO-PEG-bLF suppressed tumor growth more efficiently than free bLF (*p* < .01) and the controls (*p* < .001). No significant difference in body weight was observed between the groups until the end of the 6th week. More tumor inhibitory effect of CGO-PEG-bLF compared to free bLF could be attributed to its long circulation time due to enhanced stability of protein, more cellular uptake, and higher cytotoxic effect. No antitumor activity of control indicated that the CGO-PEG acts only as a carrier and has no cytotoxic itself. Reports have been shown that the cytotoxicity of GO is dependent on its concentration and exposure time (Chen et al., 2014; Xu et al., 2015; Zhu et al., 2017). Moreover, it seems that type of the treated cells, size and the functional groups of GO may be effective on its cytotoxicity.

## Conclusions

In this work, PEGylated GO as a drug carrier was successfully prepared in nanoscale dimensions. For enhancing the biocompatibility, solubility, and stability of GO in physiological conditions, more aminated-PEG molecules were covalently grafted (through amide bonds) on the surface of GO after carboxylation of its oxygen-containing molecules. This carrier was then successfully conjugated with bLF. In the final complex, bLF acts as an anticancer agent as well as a targeting molecule. The anticancer efficiency of the final product was evaluated *in vitro* and *in vivo*. In both situations, more anti-cancer and tumor growth inhibitory effects were obtained for bLF in conjugated form. Bovine LF as conjugated with CGO-PEG, was more uptook by the TC-1 lung cancer cells and showed significantly higher cytotoxicity against these cells through enhancement of apoptosis and disturbing the cell cycle progression. Our findings highlight the usefulness of GO-based nanocarrier as a promising drug delivery system for enhancing the therapeutic efficacy of anticancer drugs.

## References

[CIT0001] Akbari A, Akbarzadeh A, Rafiee Tehrani M, et al. (2020). Development and characterization of nanoliposomal hydroxyurea against BT-474 breast cancer cells. Adv Pharm Bull 10:39–45. 32002360 10.15171/apb.2020.005 PMC6983993

[CIT0002] Amiri B, Ahmadvand H, Farhadi A, et al. (2018). Delivery of vinblastine-containing niosomes results in potent *in vitro*/*in vivo* cytotoxicity on tumor cells. Drug Dev Ind Pharm 44:1371–6. 29532687 10.1080/03639045.2018.1451880

[CIT0003] Arias M, Hilchie A, Haney EF, et al. (2017). Anticancer activities of bovine and human lactoferricin-derived peptides. Biochem Cell Biol 95:91–8. 28165293 10.1139/bcb-2016-0175

[CIT0004] Bao H, Pan Y, Ping Y, et al. (2011). Chitosan-functionalized graphene oxide as a nanocarrier for drug and gene delivery. Small 7:1569–78. 21538871 10.1002/smll.201100191

[CIT0005] Chea C, Miyauchi M, Inubushi T, et al. (2018). Molecular mechanism of inhibitory effects of bovine lactoferrin on the growth of oral squamous cell carcinoma. PLoS One 13:e0191683. 29381751 10.1371/journal.pone.0191683 PMC5790278

[CIT0006] Chen J, Liu H, Zhao C, et al. (2014). One-step reduction and PEGylation of graphene oxide for photothermally controlled drug delivery. Biomaterials 35:4986–95. 24656608 10.1016/j.biomaterials.2014.02.032

[CIT0007] Chiani M, Shokrgozar MA, Azadmanesh K, et al. (2017). Preparation, characterization, and *in vitro* evaluation of bleomycin-containing nanoliposomes. Chem Biol Drug Des 89:492–7. 27637429 10.1111/cbdd.12869

[CIT0008] Dasari S, Samy A, Narvekar P, et al. (2018). Polygodial analog induces apoptosis in LNCaP prostate cancer cells. Eur J Pharmacol 828:154–62. 29572068 10.1016/j.ejphar.2018.03.029 PMC5918418

[CIT0009] EFSA. (2012). Scientific opinion on bovine lactoferrin. EFSA J 10:2701–27. 10.2903/j.efsa.2012.2701 PMC13151843 42112197

[CIT0010] Emadi F, Amini A, Gholami A, et al. (2017). Functionalized graphene oxide with chitosan for protein nanocarriers to protect against enzymatic cleavage and retain collagenase activity. Sci Rep 7:42258. 28186169 10.1038/srep42258 PMC5301474

[CIT0011] García-Montoya IA, Cendón TS, Arévalo-Gallegos S, et al. (2012). Lactoferrin a multiple bioactive protein: an overview. Biochim Biophys Acta 1820:226–36. 21726601 10.1016/j.bbagen.2011.06.018 PMC7127262

[CIT0012] Gibbons J, Kanwar J, Kanwar R. (2015). Iron-free and iron-saturated bovine lactoferrin inhibit survivin expression and differentially modulate apoptosis in breast cancer. BMC Cancer 15:425. 25998617 10.1186/s12885-015-1441-4 PMC4440599

[CIT0013] Guedes JP, Pereira CS, Rodrigues LR, et al. (2018). Bovine milk lactoferrin selectively kills highly metastatic prostate cancer PC-3 and osteosarcoma MG-63 cells *in vitro*. Front Oncol 8:200. 29915723 10.3389/fonc.2018.00200 PMC5994723

[CIT0014] Hashemi M, Yadegari A, Yazdanpanah G, et al. (2016). Functionalized R9-reduced graphene oxide as an efficient nanoCarrier for hydrophobic drug delivery. RSC Adv 6:74072–84.

[CIT0015] Hayes TG, Falchook GS, Varadhachary A. (2010). Phase IB trial of oral talactoferrin in the treatment of patients with metastatic solid tumors. Invest New Drugs 28:156–62. 19238327 10.1007/s10637-009-9233-9

[CIT0016] Jonasch E, Stadler WM, Bukowski RM, et al. (2008). Phase 2 trial of talactoferrin in previously treated patients with metastatic renal cell carcinoma. Cancer 113:72–7. 18484647 10.1002/cncr.23519

[CIT0017] Kazempour M, Namazi H, Akbarzadeh A, et al. (2019). Synthesis and characterization of PEG-functionalized graphene oxide as an effective pH-sensitive drug carrier. Artif Cells Nanomed Biotechnol 47:90–4. 30663418 10.1080/21691401.2018.1543196

[CIT0018] Li D, Muller MB, Gilje S, et al. (2008). Processable aqueous dispersions of graphene nanosheets. Nat Nanotechnol 3:101–5. 18654470 10.1038/nnano.2007.451

[CIT0019] Li D, Sakashita S, Morishita Y, et al. (2011). Binding of lactoferrin to IGBP1 triggers apoptosis in a lung adenocarcinoma cell line. Anticancer Res 31:529–34. 21378334

[CIT0020] Li L, Tang FQ, Liu HY, et al. (2010). *In vivo* delivery of silica nanorattle encapsulated docetaxel for liver cancer therapy with low toxicity and high efficacy. ACS Nano 4:6874–82. 20973487 10.1021/nn100918a

[CIT0021] Liu Z, Robinson JT, Sun X, et al. (2008). PEGylated nanographene oxide for delivery of water-insoluble cancer drugs. J Am Chem Soc 130:10876–7. 18661992 10.1021/ja803688x PMC2597374

[CIT0022] Lu T, Nong Z, Wei L, et al. (2020). Preparation and anti-cancer activity of transferrin/folic acid double-targeted graphene oxide drug delivery system. J Biomater Appl 35:15–27. 32202183 10.1177/0885328220913976

[CIT0023] Onishi H, Machida Y, Koyama K. (2007). Preparation and *in vitro* characteristics of lactoferrin-loaded chitosan microparticles. Drug Dev Ind Pharm 33:641–7. 17613028 10.1080/03639040601085334

[CIT0024] Pan S, Qi Z, Li Q, et al. (2019). Graphene oxide-PLGA hybrid nanofibres for the local delivery of IGF-1 and BDNF in spinal cord repair. Artif Cells Nanomed Biotechnol 47:650–63. 10.1080/21691401.2019.1575843 30829545

[CIT0025] Qiang M, Pang X, Ma D, et al. (2020). Effect of membrane surface modification using chitosan hydrochloride and lactoferrin on the properties of astaxanthin-loaded liposomes. Molecules 25:610. 10.3390/molecules25030610 PMC7036813 32019205

[CIT0026] Singh K, Srivastava G, Talat M, et al. (2015). α-Amylase immobilization onto functionalized graphene nanosheets as scaffolds: its characterization, kinetics and potential applications in starch based industries. Biochem Biophys Rep 3:18–25. 29124165 10.1016/j.bbrep.2015.07.002 PMC5668679

[CIT0027] SreeHarsha N, Maheshwari R, Al-Dhubiab BE, et al. (2019). Graphene-based hybrid nanoparticle of doxorubicin for cancer chemotherapy. Int J Nanomedicine 14:7419–29. 31686814 10.2147/IJN.S211224 PMC6751552

[CIT0028] Stankovich S, Dikin DA, Dommett GH, et al. (2006). Graphene-based composite materials. Nature 442:282–6. 16855586 10.1038/nature04969

[CIT0029] Tsuda H, Kozu T, Iinuma G, et al. (2010). Cancer prevention by bovine lactoferrin: from animal studies to human trial. Biometals 23:399–409. 20407806 10.1007/s10534-010-9331-3

[CIT0030] Tsuda H, Sekine K, Fujita K, et al. (2002). Cancer prevention by bovine lactoferrin and underlying mechanisms-a review of experimental and clinical studies. Biochem Cell Biol 80:131–6. 11908637 10.1139/o01-239

[CIT0031] Villanueva PJ, Martinez A, Baca ST, et al. (2018). Pyronaridine exerts potent cytotoxicity on human breast and hematological cancer cells through induction of apoptosis. PLoS One 13:e0206467. 30395606 10.1371/journal.pone.0206467 PMC6218039

[CIT0032] Wang B, Timilsena YP, Blanch E, et al. (2019). Lactoferrin: structure, function, denaturation and digestion. Crit Rev Food Sci Nutr 59:580–96. 28933602 10.1080/10408398.2017.1381583

[CIT0033] Wang C, Feng L, Yang H, et al. (2012). Graphene oxide stabilized polyethylene glycol for heat storage. Phys Chem Chem Phys 14:13233–8. 22914763 10.1039/c2cp41988b

[CIT0034] Xu H, Fan M, Elhissi AM, et al. (2015). PEGylated graphene oxide for tumor-targeted delivery of paclitaxel. Nanomedicine 10:1247–62. 25955123 10.2217/nnm.14.233

[CIT0035] Xu XX, Jiang HR, Li HB, et al. (2010). Apoptosis of stomach cancer cell SGC-7901 and regulation of Akt signaling way induced by bovine lactoferrin. J Dairy Sci 93:2344–50. 20494139 10.3168/jds.2009-2926

[CIT0036] Xu Z, Wang S, Li Y, et al. (2014). Covalent functionalization of graphene oxide with biocompatible poly(ethylene glycol) for delivery of paclitaxel. ACS Appl Mater Interfaces 6:17268–76. 25216036 10.1021/am505308f

[CIT0037] Xu Z, Zhu S, Wang M, et al. (2015). Delivery of paclitaxel using PEGylated graphene oxide as a nanocarrier. ACS Appl Mater Interfaces 7:1355–63. 25546399 10.1021/am507798d

[CIT0038] Yamada Y, Sato R, Kobayashi S, et al. (2008). The antiproliferative effect of bovine lactoferrin on canine mammary gland tumor cells. J Vet Med Sci 70:443–8. 18525164 10.1292/jvms.70.443

[CIT0039] Yang K, Feng L, Liu Z. (2015). The advancing uses of nano-graphene in drug delivery. Expert Opin Drug Deliv 12:601–12. 25466364 10.1517/17425247.2015.978760

[CIT0040] Zaaba NI, Foo KL, Hashim U, et al. (2017). Synthesis of graphene oxide using modified hummers method: solvent influence. Procedia Eng 184:469–77.

[CIT0041] Zhang Y, Lima CF, Rodrigues LR. (2015). *In vitro* evaluation of bovine lactoferrin potential as an anticancer agent. Int Dairy J 40:6–15.

[CIT0042] Zhang L, Lu Z, Zhao Q, et al. (2011). Enhanced chemotherapy efficacy by sequential delivery of siRNA and anticancer drugs using PEI-grafted graphene oxide. Small 7:460–4. 21360803 10.1002/smll.201001522

[CIT0043] Zhang L, Xia J, Zhao Q, et al. (2010). Functional graphene oxide as a nanocarrier for controlled loading and targeted delivery of mixed anticancer drugs. Small 6:537–44. 20033930 10.1002/smll.200901680

[CIT0044] Zhang Y, Nicolau A, Lima CF, et al. (2014). Bovine lactoferrin induces cell cycle arrest and inhibits Mtor signaling in breast cancer cells. Nutr Cancer 66:1371–85. 25356800 10.1080/01635581.2014.956260

[CIT0045] Zhou Y, Zeng Z, Zhang W, et al. (2008). Lactotransferrin: a candidate tumor suppressor-deficient expression in human nasopharyngeal carcinoma and inhibition of NPC cell proliferation by modulating the mitogen-activated protein kinase pathway. Int J Cancer 123:2065–72. 18697201 10.1002/ijc.23727

[CIT0046] Zhu H, Zhou B, Chan L, et al. (2017). Transferrin-functionalized nanographene oxide for delivery of platinum complexes to enhance cancer-cell selectivity and apoptosis-inducing efficacy. Int J Nanomedicine 12:5023–38. 28761342 10.2147/IJN.S139207 PMC5516881

